# Signaling Pathways in Oxidative Stress-Induced Neurodegenerative Diseases: A Review of Phytochemical Therapeutic Interventions

**DOI:** 10.3390/antiox14040457

**Published:** 2025-04-12

**Authors:** Zahra Sebghatollahi, Ruchika Yogesh, Neelima Mahato, Vijay Kumar, Yugal Kishore Mohanta, Kwang-Hyun Baek, Awdhesh Kumar Mishra

**Affiliations:** 1 Department of Plant Breeding and Biotechnology, Faculty of Agricultural Sciences and Food Industries, Science and Research Branch, Islamic Azad University, Tehran 1477893855, Iran; zahrasebghatollahi@gmail.com; 2 MaTestLab Inc., 2093 Philadelphia Pike, Claymont, DE 19703, USA; ruchika@matestlabs.com; 3 School of Chemical Engineering, Yeungnam University, Gyeongsan 38541, Gyeongsangbuk-do, Republic of Korea; neelima@yu.ac.kr; 4 Department of Orthopaedic Surgery, The Johns Hopkins University School of Medicine, Baltimore, MD 21205, USA; vijaykumarcbt@gmail.com; 5 Nano-Biotechnology and Translational Knowledge Laboratory, Department of Applied Biology, School of Biological Sciences, University of Science and Technology Meghalaya, Techno City, 9th Mile, Baridua 793101, Meghalaya, India; ykmohanta@gmail.com; 6 Centre for Herbal Pharmacology and Environmental Sustainability, Chettinad Hospital and Research Institute, Chettinad Academy of Research and Education, Kelambakkam 603103, Tamil Nadu, India; 7 Department of Biotechnology, Yeungnam University, Gyeongsan 38541, Gyeongsangbuk-do, Republic of Korea

**Keywords:** reactive oxygen species, NADPH oxidase, Alzheimer’s disease, Parkinson’s disease, phytochemicals, neuroprotection, therapeutic interventions

## Abstract

Oxidative stress, a pivotal driver of neurodegenerative diseases, results from an imbalance between the generation of reactive oxygen species (ROS) and cellular antioxidant defenses. This review provides a comprehensive analysis of key oxidative stress sources, focusing on NADPH oxidase (NOX) hyperactivity and mitochondrial Uncoupling Protein (UCP) downregulation. Critically, we examine the therapeutic potential of phytochemicals in mitigating NOX-mediated ROS generation through direct enzyme inhibition, including impacts on NOX subunit assembly and gene expression. Furthermore, we explore the ability of phytochemicals to bolster cellular antioxidant defenses by activating the Kelch-like ECH-associated protein 1 (KEAP1)/nuclear factor erythroid 2-related factor 2 (Nrf2)/antioxidant response element (ARE) signaling pathway, elucidating the upregulation of antioxidant genes, such as GPx, SOD, CAT, and HO-1. This review expands beyond confined overviews; emphasizes specific molecular interactions between phytochemicals and target proteins, including NOX isoforms; and provides an in-depth analysis of the specific antioxidant genes upregulated via Nrf2. This approach aims to pave the way for targeted and translatable therapeutic strategies in neurodegenerative diseases. Ultimately, this review illuminates the intricate molecular dynamics of oxidative stress in neurodegenerative diseases; underscores the potential of phytochemicals to restore redox homeostasis and reverse pathological conditions through precise modulation of key signaling pathways.

## 1. Introduction

Molecules containing at least one unpaired electron are termed free radicals, and any free radical involving oxygen is classified as a reactive oxygen species (ROS). ROS, including superoxide radicals (O^−•^), hydrogen peroxide (H_2_O_2_), and hydroxyl radicals (^•^OH), are natural byproducts of cellular metabolism [1]. While ROS at physiological levels act as crucial signaling molecules involved in various cellular processes, an imbalance between the formation of ROS and the cellular antioxidant defense system results in oxidative stress [2,3]. This state of oxidative stress, characterized by an overabundance of ROS, can damage cellular components, such as lipids, proteins, and DNA [4]. Several studies have highlighted the intricate role of ROS in neurodegenerative diseases (NDDs) [5,6]. Oxidative stress, along with dysregulation of intracellular calcium (Ca^2+^) homeostasis, has been identified as a key factor contributing to the pathogenesis of several neurodegenerative conditions, and elevated levels of ROS and Ca^2+^ have been proposed as potential diagnostic biomarkers [7,8]. Furthermore, oxidative stress can activate inflammatory signaling pathways, including tumor necrosis factor (TNF), nuclear factor kappa B (NF-κB), and mitogen-activated protein kinases (MAPKs) pathways [9,10], thereby exacerbating neuroinflammation and contributing to the progression of neurodegenerative diseases, such as Alzheimer’s disease (AD), Parkinson’s disease (PD), amyotrophic lateral sclerosis (ALS), Huntington’s disease (HD), and multiple sclerosis (MS) [11,12]. For example, in AD, ROS-mediated oxidative damage and neuroinflammation are implicated in amyloid-beta plaque formation and tau protein hyperphosphorylation [13,14]. In PD, oxidative stress contributes to the degeneration of dopaminergic neurons in the substantia nigra [15,16]. Similarly, in ALS, ROS-induced damage to motor neurons plays a crucial role in disease progression [17,18]. In amyotrophic lateral sclerosis (ALS) and frontotemporal dementia (FTD), the abnormal accumulation of TAR DNA-binding protein 43 (TDP-43) disrupts mitochondrial function, leading to excessive generation of ROS. Elevated levels of ROS further exacerbate TDP-43 misfolding and aggregation, creating a vicious cycle where the protein aggregates promote more ROS production [19,20]. Understanding the complex interplay between ROS, calcium dysregulation, and neuroinflammation is crucial for developing effective therapeutic strategies for these debilitating conditions.

## 2. Hyperactivity of NADPH Oxidase in Neurodegenerative Diseases

Hyperactivity of NADPH oxidase (NOX) has been increasingly implicated in the pathogenesis of neurodegenerative diseases. NOX enzymes, a family of multi-component enzymes, are crucial for ROS production, playing a vital role in host defense against pathogens [12]. While primarily known for their expression in phagocytic cells, NOXs are also found in non-phagocytic cells, including neurons and glial cells [21]. These enzymes catalyze the conversion of molecular oxygen to superoxide (O_2_^−•^), a precursor to other ROS [22]. Unlike other ROS-generating systems, NOX’s primary function is ROS production, making it a potential therapeutic target [23].

The prototypical NOX2 enzyme, found in phagocytes, consists of membrane-bound subunits, including the catalytic subunit gp91^phox^ (now known as NOX2) and the regulatory subunit p22^phox^. Cytosolic subunits, such as p47^phox^, p67^phox^, p40^phox^, and the small GTPase Rac, regulate the assembly and activation of the enzyme complex [24,25]. Under physiological conditions, NOX-derived ROS plays a crucial role in cell signaling, regulating processes like proliferation, differentiation, and migration [26]. However, NOX hyperactivity leads to excessive ROS production, causing oxidative stress and damage to cellular components like lipids, proteins, and DNA [27]. This oxidative stress is a significant pathological factor in neurodegenerative diseases, such as AD, PD, HD, ALS, and MS [28].

**Figure 1:**
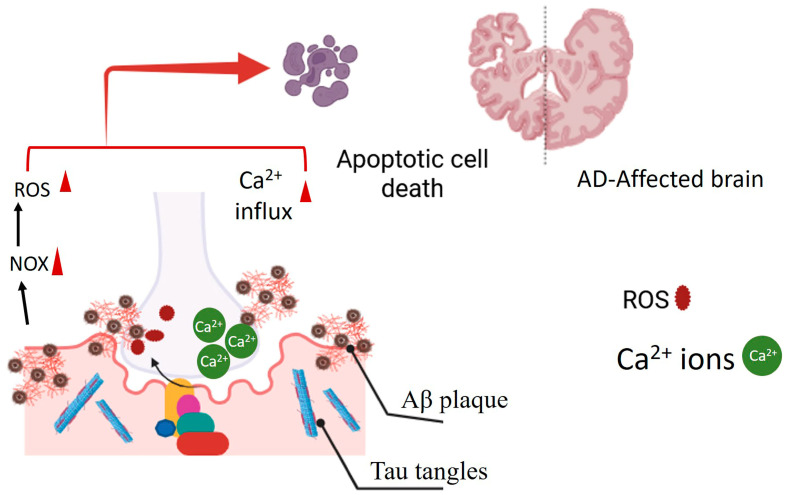
The presence of Aβ protein plaques in Alzheimer’s disease (AD) is linked to the persistent activation of the NOX enzyme. This sustained activation results in excessive production of ROS. Additionally, tau tangles can disrupt the integrity of the plasma membrane, triggering calcium influx into neural cells. This initiates apoptotic cell death in the AD-afflicted brain.

AD is characterized by a complex interplay of pathological processes involving oxidative stress, impaired signaling pathways, and protein aggregation. Amyloid-beta (Aβ) plaques, a hallmark of AD, trigger sustained NOX activation, leading to excessive ROS production and neuronal apoptosis (Figure 1) [29,30]. Notably, increased NOX2 expression has been observed in the post-mortem frontal cortex of AD patients, particularly in reactive astrocytes and microglia, linking NOX2 upregulation to neuroinflammation [31]. Furthermore, tau pathology, another key feature of AD, can disrupt membrane integrity, leading to calcium influx and subsequent NOX-mediated ROS production [24]. Recent research has focused on identifying specific NOX isoforms involved in AD and exploring the potential for isoform-targeted therapies [32,33]. Simultaneously, the phosphatidylinositol 3-kinase (PI3K)/Protein kinase B (Akt) signaling pathway, critical for neuronal survival, is consistently downregulated in AD. This diminished activity renders neurons more vulnerable to Aβ toxicity and tau hyperphosphorylation, impairing survival mechanisms and exacerbating apoptosis and synaptic deterioration. Astragalin, a natural compound, has shown neuroprotective effects in APP/PS1 mice by activating the PI3K/Akt pathway and upregulating mTOR-mediated autophagy. The latter is a key cellular clearance process [29]. Additionally, Protein Kinase C (PKC) plays a multifaceted role in AD, particularly in Aβ protein processing. Modulation of PKC activity by phytochemicals has emerged as a promising therapeutic avenue, as appropriate PKC regulation can influence Aβ protein processing and reduce their accumulation [30]. Taken together, these findings highlight the interconnection of NOX, PI3K/Akt, and PKC signaling in AD pathogenesis, suggesting that therapeutic strategies targeting multiple pathways may offer significant benefits.

PD pathogenesis is significantly influenced by oxidative stress, with NOX-derived ROS playing a critical role in dopaminergic neuron degeneration [34]. Research indicates that inhibiting NOX enzymes can reduce the accumulation of aggregated phosphorylated α-synuclein, a key pathological feature of PD, within the striatum and ventral midbrain [35]. Furthermore, recent studies also highlight the involvement of NOX in mitochondrial dysfunction, a central factor in PD pathogenesis [36]. Concurrently, the PI3K/Akt signaling pathway, essential for dopaminergic neuron survival, is frequently disrupted in PD. Oxidative stress, α-synuclein accumulation, and other pathological factors impede PI3K/Akt signaling, accelerating neuronal death. Conversely, maintaining or enhancing the Akt activity has demonstrated protective effects on these vulnerable neurons [31]. These findings underscore the interconnected roles of NOX, mitochondrial function, α-synuclein, and PI3K/Akt signaling in PD, suggesting that therapeutic strategies targeting multiple pathways may offer enhanced neuroprotection.

In ALS, NOX knockout in glial cells, combined with the NOX inhibitor apocynin, extended the lifespan of mice in an ALS model [32]. Research has investigated the specific NOX isoforms expressed in ALS-affected motor neurons and the potential for selective inhibition [33]. Mitochondria, the primary source of ROS, are particularly susceptible to damage in ALS. The accumulation of misfolded TDP-43 within mitochondria disrupts their function, increasing ROS production and initiating a vicious cycle of oxidative stress and neurodegeneration [19,20].

HD pathogenesis involves a complex interplay of oxidative stress mechanisms. Mutant huntingtin protein (mHTT) not only directly interferes with PI3K/Akt signaling, leading to neuronal dysfunction, increased susceptibility to excitotoxicity, and neuronal atrophy [34], but also induces oxidative stress by impairing mitochondrial function and enhancing NOX activity [35]. Consequently, NOX-derived reactive oxygen species (ROS) contribute significantly to the oxidative damage observed in HD, and research is actively exploring the interactions between NOX-mediated ROS production and other oxidative stress pathways within the disease [36]. This multifaceted impact of mHtt underscores the importance of targeting multiple oxidative stress pathways, including NOX and PI3K/Akt, in developing effective therapeutic strategies for HD.

In multiple sclerosis (MS), increased microglial and macrophage activation, associated with NOX subunit upregulation, leads to excessive ROS generation, contributing to demyelination [37]. Research has focused on the role of NOX2 in microglia activation and its contribution to neuroinflammation in MS [38].

Disruptions in the PI3K/Akt signaling pathway, a key regulator of neuronal survival, growth, and metabolism, are implicated in the pathogenesis of AD, PD, and HD. Phytochemicals, including curcumin, resveratrol, berberine, and quercetin, demonstrate the ability to modulate this pathway and offer potential therapeutic benefits. Curcumin promotes neuronal survival and reduces oxidative stress by directly activating PI3K/Akt. Resveratrol, by enhancing SIRT1 activity, indirectly activates PI3K/Akt, providing neuroprotection. Berberine safeguards neurons against oxidative stress, inflammation, and apoptosis through the combined activation of PI3K/Akt and Nrf2. Furthermore, quercetin’s modulation of PKC signaling pathways suggests a role in mitigating neurodegenerative processes [39].

In summary, NOX enzymes are thus central players in chronic central nervous system disorders and neurodegeneration. NOX enzymes are increasingly recognized as critical therapeutic targets [32,40]. Current research is actively exploring strategies to modulate NOX activity, including the development of specific NOX inhibitors, targeting regulatory subunits, and manipulating upstream signaling pathways. However, while phytochemicals offer promising avenues for regulating oxidative stress through NOX modulation, existing research often lacks detailed mechanistic insights and consistent in vivo validation. Studies frequently focus on general antioxidant effects, overlooking the nuanced molecular interactions between phytochemicals and specific NOX isoforms. Furthermore, variations in experimental design, dosage, and bioavailability impede the translation of these findings into clinical applications. This review aims to bridge these gaps by providing a comprehensive synthesis of the intricate molecular mechanisms through which phytochemicals modulate NOX activity. We explore both transcriptional and post-translational regulatory pathways and examine: (a) the inhibition of NOX subunit assembly (e.g., apocynin’s interference with p47^phox^ translocation); (b) the suppression of NOX gene expression (e.g., quercetin’s impact on p47^phox^); (c) the disruption of upstream signaling pathways (e.g., PKC, MAPK, PI3K/Akt); (d) the modulation of transcription factors (e.g., NF-κB, AP-1); and (e) the direct scavenging of reactive oxygen species (ROS), potentially altering NOX subunit conformation (e.g., epigallocatechin gallate (EGCG) metabolites). Additionally, we explore isoform-specific inhibitory effects, such as those observed with resveratrol and fucoidan, potentially through competition with NADPH binding or disruption of electron transfer. This review elucidates these specific molecular-level interactions and aims to provide a more refined understanding of phytochemical-mediated NOX modulation, thereby facilitating the objective of the development of targeted and clinically translatable therapeutic strategies.

### Therapeutic Effects of Phytochemicals Through NADPH Oxidase Inhibition

Several phytochemicals have demonstrated therapeutic potential in neurodegenerative diseases by targeting NOX activity and mitigating oxidative stress. Apocynin, a naturally occurring acetovanillone that can be isolated from the medicinal plant, *Jatropha multifidi*, is known for its ability to attenuate the hyperactivity of NADPH oxidase, leading to a significant reduction in the production of superoxide radicals [41,42]. Metabolically active apocynin, also referred to as diapocynin, can selectively inhibit the assembly of NADPH oxidase, showing a significant ameliorative effect on oxidative stress. Its oral administration has been demonstrated to prevent the progression of AD, PD, HD, and ALS in animal models [41].

Quercetin, a flavonoid, is commonly found in a variety of plants, including berries, grapes, citrus fruits, and onions. It can be efficiently extracted from onion skin waste (*Allium cepa* L.) [43,44]. This compound exerts a suppressive effect on the expression of the NADPH oxidase subunit P47^phox^. As a result, this regulatory action leads to the inhibition of NADPH oxidase activity, ultimately reducing the production of superoxide radicals [45]. Reducing the production of ROS can serve as a protective measure against mitochondrial dysfunction, thereby preserving cytochrome c and inhibiting caspase activation. This, in turn, helps mitigate neuroinflammation and prevents apoptotic cell death in neural tissue. The management of oxidative stress through the use of quercetin has shown a protective effect against neurodegenerative conditions such as AD, PD, and HD [46].

Aβ protein plaques and neurofibrillary tangles have been observed to activate NADPH oxidase in transformed microglial cells. This activation can result in the overproduction of ROS, including peroxynitrite [47]. Curcumin, the bioactive compound isolated from turmeric root (*Curcuma longa*), a commonly utilized spice in Indian cuisine, has been found effective in scavenging a variety of ROS by (a) inhibiting ROS-generating enzymes, such as LOX, COX, and xanthene oxidase; (b) enhancing the antioxidant enzymatic activity of SOD, CAT, GPx, and OH-1; (c) increasing GSH levels via upregulation of glutathione transferase and their mRNAs; and (d) peroxyl radical scavenging due to its lipophilic nature, also known for its chain-breaking antioxidant activity [48]. Furthermore, it demonstrates pleiotropic effects, possesses the potential to suppress microglial transformation, and decreases the production of ROS by inhibiting NADPH oxidase. As a result, it presents a promising preventive measure against a broad range of neurodegenerative diseases, including AD, PD, HD, ALS, and MS [47,49]. Curcumin demonstrates an affinity for binding to aggregated proteins, specifically Aβ and tau, within neuronal cells. This interaction is associated with the modulation of their clearance mechanisms in the context of AD. Consequently, it emerges as a compelling candidate for therapeutic intervention in AD [50].

Epigallocatechin-3-gallate (EGCG) and its metabolites have been empirically established to exert a pronounced reduction in NADPH oxidase activity, thus affording a substantial amelioration of oxidative stress [51]. To elaborate on that, its biosynthetic metabolite, 3′-O-methyl-epicatechin-5-O-glucuronide (a flavan-3-ol, found in tea, cocoa, red wine, and apple) has shown promising protective effects against oxidative stress and mitochondrial damage. This suggests its potential as a therapeutic substance for the treatment of AD [52,53]. Moreover, their bioactive metabolites have been discerned as key contributors to the substantial amelioration of neuroinflammation and the prevention of apoptotic cell death, notably within the striatal region of the brain in the context of PD and HD [54,55].

Resveratrol is a polyphenolic compound that is abundant in the skin of dark grapes (*Vitis vinifera*) and serves as a prominent constituent of red wine [56]. It has exhibited noteworthy inhibitory activity against NADPH oxidase, consequently reducing the subsequent generation of ROS [57]. Furthermore, it has shown promise in diminishing the activation of reactive microglia and the treatment of neuroinflammation, a key factor in the advancement of various neurodegenerative conditions [58].

Fucoidan, a polysaccharide comprising L-Fucose and sulfate ester moieties, is isolated and purified from brown seaweed (Phaeophyceae) and the cell walls of brown algae. This plant-derived polysaccharide has demonstrated an inhibitory effect on NADPH oxidase-1 and the generation of ROS [59,60]. Furthermore, it has demonstrated a neuroprotective effect, potentially reversing the neurodegenerative processes associated with PD and mitigating brain injury. These observations indicate that fucoidan holds promise as a strong candidate for drug development in the realm of neurodegenerative diseases [61,62]. A detailed list of NADPH oxidase inhibitory phytochemicals is provided in Table 1.

## 3. Downregulation of Mitochondrial UCPs in Neurodegenerative Diseases

Mitochondria are indispensable cellular organelles tasked with facilitating cellular respiration and the synthesis of ATP, a pivotal energy carrier molecule. Nonetheless, impaired mitochondrial function can lead to an excessive production of ROS and subsequent oxidative stress [63]. Consequently, this mitochondrial dysfunction results in damage to cellular components, including lipid peroxides, misfolded proteins, and double-strand breaks in DNA, which are recognized as fundamental causative factors in the development of neurodegenerative diseases [64,65,66].

**Figure 2:**
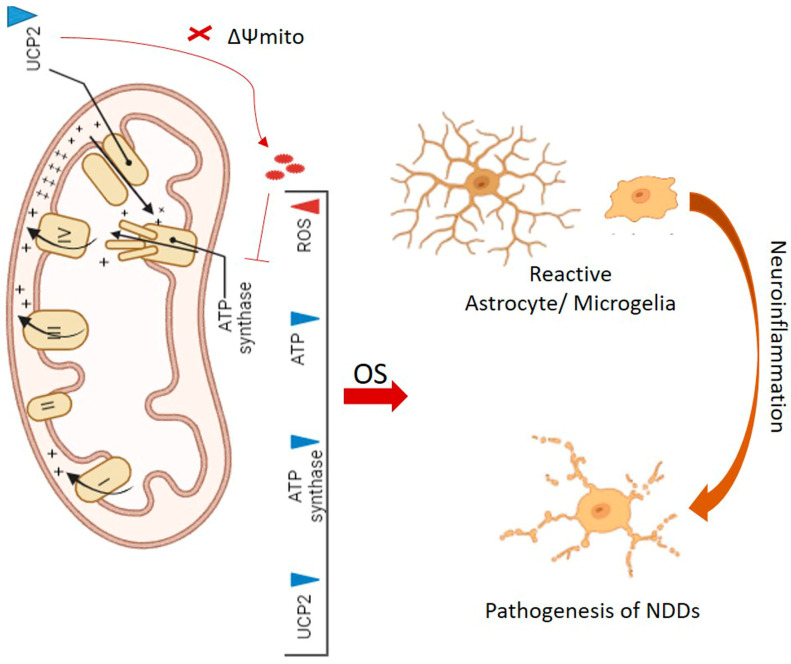
The downregulation of UCP2 results in the dissipation of mitochondrial membrane potential (ΔΨmito) and accumulation of ROS, which subsequently inhibits the activity of ATP synthase. Overall, the consequence is a decrease in the activities of UCP2, ATP synthase, ATP production, and an excessive accumulation of ROS. The latter can induce OS and the formation of reactive astrocyte/microglial cells. This interplay stands as a significant factor in neuroinflammation and the pathogenesis of neurodegenerative diseases (NDDs).

Mitochondrial uncoupling proteins (UCPs) are members of the mitochondrial anion transporter family, residing within the inner mitochondrial membrane. They have been characterized for their role in regulating cellular homeostasis, encompassing a spectrum of functions, from thermogenesis to the modulation of ROS generation [67]. UCPs, integral to mitochondrial function, hold the remarkable ability to regulate the mitochondrial inner membrane potential (ΔΨm), thus affecting cellular bioenergy dynamics. By efficiently dissipating energy, such as heat, UCPs become central players in cellular redox signaling pathways, orchestrating intricate metabolic responses [68]. The re-entry of protons from the inner membrane space to the mitochondrial matrix, a process intricately tied to proton motive force, leads to a reduction in the proton gradient directed towards the matrix. This, in turn, precipitates the dissipation of the Δψm. Consequently, this reduction in Δψm translates to a decreased production of mitochondrial ROS, showcasing the interconnectedness of mitochondrial bioenergy with cellular redox homeostasis (Figure 2) [69,70]. The dysregulation of UCPs has emerged as a pivotal factor in the pathogenesis of various diseases [71]. In this context, the overexpression of Uncoupling protein 2 (UCP2) and the ensuing reduction in mitochondrial ROS levels have been observed in cancer cells. This phenomenon can trigger pro-survival signaling pathways, potentially contributing to the immortalization or chemoresistance of cancer cells [72]. On the contrary, the downregulation of UCP2 results in heightened ROS production and compromised mitochondrial function. This detrimental cascade can culminate in the development of cardiovascular diseases and eventual heart failure [73,74]. Furthermore, reduced UCP2 expression has been linked to the advancement and emergence of neurodegenerative conditions (Figure 2) [75,76]. The distinctive expression patterns of UCP2/4/5 are evident within cerebral cells. Their reduced expression levels have been empirically associated with elevated production of ROS, thereby underscoring their indispensable role in preserving cellular redox homeostasis. Given their notable biological significance, UCPs emerge as compelling molecular targets for the mitigation of neurodegenerative conditions [71].

Within microglial cells, UCP2 serves as a pivotal guardian, playing a significant role in the negative regulation of ROS production. UCP2 is crucial for maintaining mitochondrial membrane potential, calcium homeostasis, and mitigating ROS levels. These functions collectively contribute to its role in safeguarding against neuronal cell death and the onset of neurodegenerative diseases (NDDs), such as AD and PD [71].

A reduction in UCP2 levels can lead to the accumulation of ROS and initiation of a phenotypic switch in both astrocytes and microglial cells. The latter promotes their transition to an active state. This transition can subsequently escalate neuroinflammation and contribute to the progression of neurodegenerative diseases (Figure 2) [77]. Neuroinflammation in the hippocampus has demonstrated a strong correlation with decreased UCP2 expression in activated microglial cells. This relationship results in oxidative damage and apoptotic neuronal cell death, ultimately leading to impaired short- and long-term memory in a mouse model of cognitive decline [78].

The treatment of melatonin in rat primary microglia cultures has been observed to preserve mitochondrial function by upregulating mitochondrial UCP2 within the activated microglia. This phenomenon leads to a reduction in the generation of ROS and a subsequent decrease in neuroinflammation. The recognized pivotal role of mitochondrial UCP2 in modulating neuroinflammation highlights its status as a promising therapeutic target for the treatment of neurodegenerative diseases [79].

## 4. Cellular Antioxidant Defense Mechanisms

ROS are generated as by-products of normal cellular metabolism. However, their accumulation can give rise to detrimental effects, including lipid peroxidation, protein dysfunction, and single/double-strand breaks in DNA [80]. Precise regulation of redox homeostasis stands as a critical factor in sustaining cellular physiological equilibrium and acting as a barrier against the onset of oxidative stress-induced neurodegenerative diseases [81].

The paramount functions of the cellular antioxidant system encompass maintaining cellular redox homeostasis and safeguarding against the perils of oxidative stress. This intricate system plays a pivotal role in counteracting the emergence of oxidative stress-induced neuroinflammation. Numerous studies have provided evidence pointing to the dysregulation of this redox homeostasis system, implicating it in the pathogenesis of neurodegenerative diseases [82,83,84]. Activation of cellular antioxidant defense mechanisms, primarily orchestrated through the KEAP1/Nrf2/ARE signaling pathway, holds the capacity to usher in cellular redox homeostasis. By doing so, it not only guards against oxidative damage but also halts the progression of neurodegenerative diseases [85].

**Figure 3:**
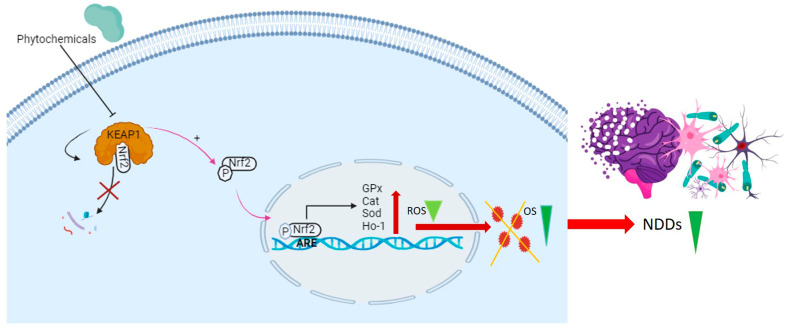
This diagram illustrates the disruption of the KEAP1-Nrf2 complex by phytochemicals, preventing the degradation of Nrf2. This disruption results in the liberation of Nrf2 in the form of phosphorylated Nrf2 (p-Nrf2), which is then translocated to the nucleus. In the nucleus, p-Nrf2 binds to antioxidant response elements (AREs) in the promoter region, influencing the expression of key antioxidant genes (GPx, CAT, SOD, HO-1). This process reduces ROS and alleviates OS, thereby mitigating NDDs.

Cells have evolved a sophisticated antioxidant defense mechanism, achieved through the upregulation of key antioxidant enzymes. These enzymes encompass superoxide dismutase (SOD), glutathione peroxidase (GPx), catalase (CAT), and hem oxygenase-1 (HO-1) [86,87]. This antioxidant defense mechanism has been demonstrated to be upregulated through the Nrf2/ARE axis (Figure 3) [88].

SOD is a vital antioxidant enzyme that incorporates metal-link moieties of zinc, copper, and manganese. It is distributed across various cellular compartments, including the cytosol, nucleus, and mitochondrial matrix [89]. The primary function of this enzyme is to catalyze the conversion of superoxide radicals into oxygen and hydrogen peroxide. Notably, decreased levels of SOD enzyme have been reported in neural cells during the advanced stages of Alzheimer’s [90].

CAT is an oxidative enzyme primarily located in peroxisomes within most aerobic cells. Its main function involves catalyzing the conversion of hydrogen peroxide into water and oxygen [91]. Reduced levels of catalase in neural cells have demonstrated a direct impact on the progression of neurodegenerative diseases, including Alzheimer’s (AD) and Parkinson’s (PD) [92,93].

GPxs belong to the class of oxidoreductase enzymes. They play a crucial role in reducing hydrogen peroxide and phospholipid hydroperoxides, thereby serving as a preventive measure against necrotic cell death in HD [94,95]. The dysregulation of GPx activity has been observed in both acute and chronic neurodegenerative diseases, such as AD and ALS. This observation positions GPx as a promising candidate for therapeutic interventions [96].

The HO-1 enzyme plays a crucial role in breaking down heme from hemoglobin, leading to the formation of its metabolites, including ferric ions, biliverdin, and carbon monoxide. Notably, the dysregulation of HO-1 activity in glial brain cells has exhibited a significant association with both AD and PD [97].

Mitochondrial antioxidant systems, including the manganese superoxide dismutase (MnSOD) enzyme and the non-enzymatic molecule glutathione (GSH), have been recognized as critical components of cellular scavenging mechanisms. They play a significant role in ameliorating the initiation and progression of neurodegenerative diseases, such as AD and PD [98].

An expanding body of evidence has firmly established that vitamin E deficiency can serve as a major risk factor in the development of neurodegenerative diseases, including AD, PD, and ALS. This correlation is primarily attributed to the antioxidant properties of vitamin E, which play a pivotal role in mitigating cellular oxidative stress [99]. Furthermore, a decline in vitamin C levels has also been linked to neurodegenerative diseases. This connection is attributed to the overproduction of peroxynitrite anions, which underscores the significance of vitamin C in the context of neurodegenerative diseases [100].

### 4.1. KEAP1/Nrf2/ARE Signaling Pathway

Under normal physiological conditions, nuclear factor erythroid 2-related factor 2 (Nrf2) remains in an inactive state within the cytoplasm, bound by its inhibitor, E3 ligase adaptor Kelch-like ECH-associated protein 1 (KEAP1) [101]. However, exposure of cells to oxidative stress triggers the activation of Nrf2, leading to its liberation from ubiquitination by KEAP1. As a result, Nrf2 is able to translocate to the nucleus [102]. Nrf2 possesses the capability to stimulate the antioxidant response element (ARE) site within gene promoters, consequently activating the transcription of genes associated with antioxidant responses. This includes pivotal enzymes like SOD, GPx, CAT, and HO-1. The activation of the KEAP1/Nrf2/ARE signaling pathway plays a pivotal role in preserving cellular redox homeostasis and serves as a protective measure against the onset of neurodegenerative diseases [103]. Similarly, investigations have revealed that mice with KEAP1 knockdown exhibit heightened levels of SOD, GPx, and CAT. This upsurge in antioxidant enzyme levels translates to a decreased susceptibility to oxidative stress. Furthermore, the attenuation of KEAP1 reinforces the significance of Nrf2 activation in bolstering antioxidant defense mechanisms. This phenomenon contributes to safeguarding cells against the impact of oxidative stress-induced neurodegenerative diseases [104].

### 4.2. Therapeutic Effects of Phytochemicals Through Nrf2 Activation

Deficiency in Nrf2 has been identified in the human brain, establishing a direct correlation with the pathogenesis of AD, PD, and ALS [105]. The activation of the KEAP1/Nrf2/ARE signaling pathway plays a pivotal role in maintaining redox homeostasis and acts as a preventive measure against neurodegenerative diseases induced by oxidative stress [106].

Remarkably, a diverse array of natural compounds, including phenylenediamines, quinones, oxidizable diphenols, thiocarbamates, trivalent arsenicals, isothiocyanates, dithiolethiones, hydroperoxides, vicinal dimercaptans, Michael acceptors, heavy metals, and polyenes, have shown the potential to activate the Nrf2 pathway [107].

**Figure 4:**
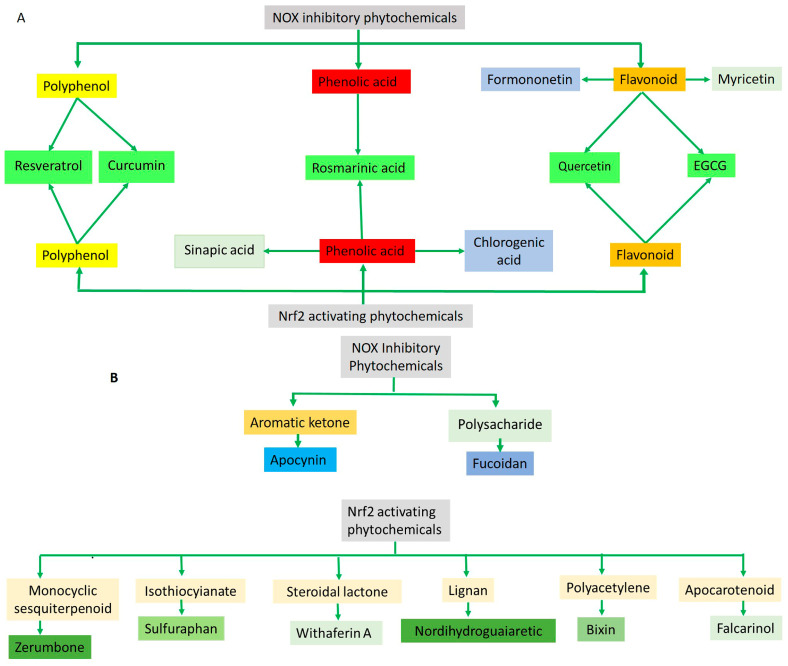
(**A**) The diagram illustrates that the majority of the phytochemicals associated with NOX inhibition and Nrf2 activation belong to the polyphenol/phenolic acid category and its subgroup, flavonoid. Notably, resveratrol, curcumin, rosmarinic acid, quercetin, and EGCG demonstrate both Nrf2 activating and NOX inhibitory activities. (**B**) Apocynin and fucoidan, derived from two distinct compound categories, are linked to NOX inhibitory activity. The remaining phytochemicals are categorized into diverse compound groups, each associated with Nrf2 activation.

Phytochemicals, which include terpenoids, alkaloids, and primarily flavonoids as phenolic compounds, have been identified as agents possessing neuroprotective properties. These compounds operate by activating the Nrf2 signaling pathway to exert their beneficial effects (Figure 4). [108]. Sulforaphane, which belongs to the category of isothiocyanates, can be extracted from broccoli (*Brassica oleracea* L.; a Brassicaceae plant) and is known for its ability to activate the Nrf2 pathway [109]. The interplay between sulforaphane and KEAP1 induces a structural alteration in KEAP1, prompting the relocation of Nrf2 to the nucleus. This process initiates the activation of the Nrf2/ARE axis, thereby enhancing the transcription of genes responsible for antioxidants. This coordinated response is strategically aimed at countering oxidative stress and the consequent progression of neurodegenerative diseases [110]. Curcumin, zerumbone, and falcarinol demonstrate the capacity to induce Nrf2 activation, mitigating oxidative stress-induced neuroinflammation and neurodegenerative diseases [111]. A detailed list of Nrf2-activating phytochemicals is provided below in Table 2.

Curcumin and its principal metabolite, hexahydrocurcumin, demonstrate an inhibitory effect on KEAP1, leading to the upregulation of the Nrf2/HO-1 signaling pathway. Additionally, it can modify and activate glutamate-cysteine ligase, a crucial enzyme involved in the production of the cellular antioxidant peptide known as glutathione [112,155]. Furthermore, this compound exhibits notable efficacy in suppressing protein aggregation within the brain, with a specific focus on α-synuclein and Aβ oligomers. Consequently, it presents significant potential as a potent therapeutic agent against PD and AD, respectively [113].

Zerumbone, a monocyclic sesquiterpenoid, is found in perennial herbs and can be extracted and purified from wild ginger (*Z. zerumbet*) [114]. It has shown inducible effects on endogenous antioxidants, such as HO-1, GCLC, GCLM, and NQO1, in microglial cells, which can be stimulated by the Nrf2/ARE axis. Therefore, it can mitigate oxidative stress and neuroinflammation-induced neurodegenerative diseases [111,115].

Falcarinol, a polyacetylene, is found in a huge number of plants, classified in the Apiaceae family, and is extractable from carrots (*D. carota*) [116]. It can induce the Nrf2/ARE axis, leading to the activation of antioxidant enzymes, including HO-1 and SOD. Furthermore, it can downregulate proinflammatory genes, including *Il-6*, *Tnf-α*, *Inf-γ*, *Il-10*, and *Stat3* [117]. This compound can mitigate mitochondrial dysfunction and oxidative damage to cellular components, as indicated by reduced levels of MDA and LDH [118].

Nordihydroguaiaretic acid has been identified in the leaves and twigs of the perennial desert shrub, and it can be extracted from the creosote bush (*L. tridentata*) [119]. It has been recognized for its ability to act as an inducer of the Nrf2/HO-1 signaling pathway. As a result, it exhibits substantial potential as a therapeutic agent against neurodegenerative diseases triggered by oxidative stress [120].

Bixin, an apocarotenoid extractable from the seeds of the achiote tree (*B. orellana*), has shown the capacity to activate the Nrf2/ARE signaling pathway [156]. This compound has exhibited a significant decrease in the serum levels of pro-inflammatory cytokines that contribute to neuroinflammation-induced neurodegenerative diseases [122].

Withaferin A, a naturally occurring steroidal lactone, can be extracted and purified from the Indian herbal medicine, “Ashwagandha”. This herb is also known as Indian Winter cherry or Indian Ginseng (*W. somnifera*) [123]. Both in silico and in vitro studies have shown that it can bind to the cysteine residue of KEAP1, thereby disrupting its interaction with Nrf2. This disruption leads to the induction of the Nrf2/HO-1 axis [124]. It has demonstrated a significant therapeutic effect in mouse models of neurodegenerative conditions in the frontotemporal lobe, such as ALS [157].

Epigallocatechin-3-gallate (EGCG), a vital bioactive compound found in green tea (*C. sinensis*), has been shown to activate the Nrf2/ARE signaling pathway. The ARE element can regulate the expression of downstream molecular pathways, leading to elevated expression levels of endogenous antioxidant genes, such as *HO-1* and *GPx* [125,126]. EGCG has the capability to directly target neuronal aggregates of Aβ protein in AD and α-synuclein protein in PD. Consequently, it has the potential to reverse the neurodegenerative processes associated with these conditions [127].

Rosmarinic acid, a phenolic compound that is abundant in the plants of the Lamiaceae plant family, can be extracted from rosemary (*R. officinalis*) [128]. It has been recognized as a stimulator of the Nrf2/ARE axis, which results in the attenuation of oxidative stress and the subsequent reduction of cellular toxins associated with mitochondrial damage, such as malondialdehyde (MDA) and DNA damage marker 8-hydroxy-2′-deoxyguanosine (8-OHdG) [129]. Numerous in vivo and clinical studies have shown that rosmarinic acid exhibits therapeutic effects in certain neurodegenerative diseases, including Alzheimer’s (AD, Parkinson’s (PD), and Huntington’s diseases (HD) [130].

Myricetin has been identified as a major constituent in plants of the Polygonaceae family, primarily isolated from birdweed (*P. suffruticosum*) cultivars [131]. It has been shown to possess the capability to activate the Nrf2/ARE signaling pathway, thereby leading to the enhancement of downstream antioxidant enzymes, including CAT, SOD, and HO-1. Furthermore, myricetin plays a role in preserving efficient mitochondrial function in neural cells and prevents apoptotic cell death in the brains of animal models with neurological deficits [132]. It demonstrates an inductive effect in promoting the detoxification of aggregated oligomers, specifically Aβ and α-synuclein. These protein aggregates are strongly associated with the development of neurodegenerative diseases, including AD, PD, HD, and amyotrophic lateral sclerosis (ALS), as supported by preclinical research [133,158].

Quercetin has the capacity to activate the Nrf2/ARE signaling pathway, which, in turn, initiates the expression of genes responsible for the cellular antioxidant system [134]. Furthermore, quercetin has been demonstrated to upregulate downstream genes, such as *HO-1*, *NQO1*, and SOD2 (MnSOD) [135]. The administration of low doses of quercetin has exhibited significant therapeutic effects in the context of neurodegenerative diseases, underscoring its potential as an alternative candidate for drug development [136].

Resveratrol has shown its ability to induce the Nrf2/HO-1 signaling pathway by inhibiting the KEAP1 protein. Moreover, it facilitates the translocation of Nrf2 into the nucleus in a mouse model of AD [137]. Moreover, it possesses the capacity to activate mitochondrial transcription factor A (mtTFA) by inducing Nrf2, thereby orchestrating mitochondrial biogenesis and reinforcing the mitochondrial antioxidant system [138,139]. Numerous in vitro and in vivo studies have suggested that quercetin may serve as a potential mitigator of oxidative stress, functioning as a protective agent against neurodegenerative diseases, such as AD, PD, ALS, and HD [138].

Formononetin is extracted and purified from legumes, as well as various types of Red Clover (*T. pratense* L.). It is a well-established phytoestrogen classified within the isoflavone category [140]. It can block the specific domain of KEAP1, thereby preventing Nrf2 ubiquitination and facilitating the induction of the Nrf2/ARE axis. This action is instrumental in maintaining cellular redox homeostasis [141]. Formononetin has demonstrated the ability to induce the elimination of Aβ plaques and inhibit Monoamine oxidase-B (MAO-B)-mediated preservation of dopaminergic neurons. These findings suggest that it could serve as an effective agent for reversing neurodegenerative diseases [142].

Sinapic acid belongs to the hydroxycinnamic acid group and is commonly obtained from sources, such as coffee, tea, and wine. It can also be isolated from bokbunja wine, which is made from Korean bramble (*R. coreanus*) [143,159]. It has been observed to induce the Nrf2/ARE signaling pathway. This induction, in turn, leads to the upregulation of crucial antioxidant genes, including *SOD*, *GPx*, *CAT*, and *HO-1* [144]. This compound demonstrates an inhibitory effect on the acetylcholinesterase enzyme, resulting in the preservation of acetylcholine neurotransmitter levels and cholinergic neurons. Additionally, it blocks cholinergic receptors, suggesting its potential to ameliorate cognitive impairment in AD and PD [145].

Chlorogenic acid, a nutritional cinnamate derivative, is present in various green and roasted coffee cultivars, with the highest content being obtainable through the extraction and purification from coffee, particularly *C. canephora* [146,147]. Chlorogenic acid has demonstrated the ability to induce the Nrf2/HO-1 antioxidant pathway, leading to the inhibition of the primary inflammatory transcription factor, nuclear factor kappa B (NF-κB). This mechanism holds promise for mitigating neuroinflammation-associated neurodegenerative diseases driven by oxidative stress [148,160].

*G. biloba* extract, abundant in flavonoids and terpenoids, is acknowledged for its neuroprotective properties [161]. It exhibits a distinct molecular mechanism involving binding to gene elements responsible for antioxidant responses, AREs, and the inhibition of the KEAP1 protein. Consequently, Nrf2 escapes proteasomal degradation facilitated by KEAP1 and translocates into the nucleus. The convergence of these molecular interactions and signaling cascades culminates in the upregulation of antioxidant enzymes, including SOD and CAT, within the hippocampus, striatum, and substantia nigra. This upregulation plays a critical role in counteracting neurodegenerative diseases [150,151].

The extract originating from *B. monnieri* encompasses a diverse array of 52 bioactive compounds, where Asiatic acid and Loliolide emerge as prominent constituents, distinguished for their notable therapeutic prospects in the context of AD [152]. It has been demonstrated that *B. monnieri* extract strengthens the endogenous antioxidant system through the Nrf2 signaling pathway, leading to the reversal of neurodegenerative conditions. This induction of downstream molecules, such as HO-1 and glutamate cysteine ligase catalytic subunit (GCLC), plays a pivotal role in disputing OS by effectively detoxifying ROS [153,154]. Numerous in vivo studies have indicated that *B. monnieri* is a well-established medicinal plant known for its efficacy in mitigating OS and exerting a beneficial impact on mitochondrial dysfunction. Consequently, it exhibits therapeutic effects on cognitive deficits in AD [162].

## 5. Conclusions

This in-depth review highlights that oxidative stress, driven by both mitochondrial dysfunction and NOX hyperactivity, is a central driver in the pathogenesis of neurodegenerative diseases. Mitochondrial UCP downregulation, particularly UCP2, leads to increased production of ROS and mitochondrial impairment, while diminished KEAP1/Nrf2/ARE signaling reduces cellular antioxidant defenses. Conversely, phytochemicals like melatonin, curcumin, EGCG, and resveratrol demonstrate therapeutic potential by regulating UCP2, activating the Nrf2/ARE pathway, restoring redox homeostasis, and preserving mitochondrial function. Moreover, compounds such as apocynin, quercetin, zerumbone, and *G. biloba* extract offer diverse neuroprotective mechanisms, including NOX inhibition, KEAP1 modulation, and direct ROS scavenging.

This review elucidates the critical roles of both NOX and mitochondrial UCPs in oxidative stress-induced neurodegeneration, emphasizing the promise of phytochemical interventions. By targeting these specific pathways, phytochemicals present novel therapeutic strategies. The intricate interplay between NOX, PI3K/Akt, and PKC signaling further underscores the need for multi-target approaches. While these findings offer significant insights, further research and clinical trials are imperative to validate these phytochemical-based strategies and translate them into effective treatments for neurodegenerative pathologies.

## Figures and Tables

**Table 1:** NADPH oxidase inhibitory phytochemicals and their molecular mechanisms.

Name of Phytochemicals and Chemical Structures	Source of Phytochemicals	Molecular Mechanisms and Type of Study	References
Apocynin (Aromatic Ketone) 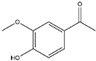	It is found in the roots of plants of the Apocynaceae family. Commonly isolated from the roots of the Coral plant (*Jatropha multifidi* L.).	Inhibits the assembly of NADPH oxidase subunits; reduces ROS generation in microglial cells, thereby preventing neurodegenerative diseases (in vivo study).	[41,42]
Quercetin (Flavonoid) 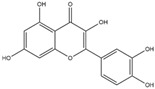	It is distributed in various fruits and vegetables such as citrus, apples, dark berries, and onions. Discarded outer layers of onions (*Allium cepa* L.) are a sustainable and economically viable source for the isolation of this bioactive compound.	Inhibits NADPH oxidase through the downregulation of the P47^phox^ subunit; mitigates reactive oxygen species (ROS) generation and mitochondrial dysfunction, thereby attenuating neuroinflammation-induced neurodegenerative diseases (in vivo and clinical studies).	[44,46]
Curcumin (Polyphenol) 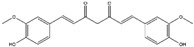	It is a bioactive compound naturally found in Indian spices, and can be isolated from the roots of turmeric, a staple in Indian cuisine (*Curcuma longa* L.)	Inhibits NADPH oxidase, suppresses microglial transformation, and reduces ROS generation, thereby preventing neurodegenerative diseases (in vivo and preclinical studies).	[49,50]
Epigallocatechin-3-gallate (EGCG) (Flavonoid) 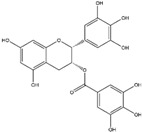	It is abundantly found in green tea and can be isolated from the leaves of the tea plant (*Cammellia sinesis* (L.) Kuntze)	Inhibits NADPH oxidase and attenuates mitochondrial dysfunction, thereby preventing OS-induced neurodegenerative diseases (in vivo study).	[55]
Resveratrol (Polyphenol) 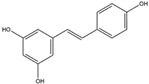	It is naturally present in nuts, berries, grapes, and red wine. It can be isolated from the skin of dark grapes, specifically from the variety (*Vitis vinifera* L.).	Inhibits NADPH oxidase and subsequent generation of reactive oxygen species (ROS); suppresses reactive microglia under neuroinflammatory conditions, contributing to the prevention of NDDs (in vivo and clinical studies).	[56,58]
Fucoidan (Polysaccharide-Containing L-Fucos and Sulfate Ester Groups) 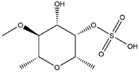	It is a bioactive compound that can be isolated from brown seaweed and the cell walls of brown algae belonging to the Phaeophyceae class.	Inhibits NADPH oxidase-1 and subsequent ROS generation, leading to improvement in neurodegenerative processes associated with brain injury associated in PD (in vivo study).	[59,61]

NDDs: neurodegenerative diseases; OS: oxidative stress.

**Table 2:** Detailed list of Nrf2-activating phytochemicals and their molecular mechanisms.

Name of Phytochemicals and Chemical Structure	Source of Phytochemicals	Molecular Mechanisms and Type of Study	References
Sulfuraphane (Isothiocyanates) 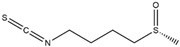	It is a bioactive compound naturally found in the plant family of Brassicaceae and can be isolated from broccoli (*Brassica oleracea* L.).	Sulforaphane can interrupt the KEAP1-Nrf2 complex through its interaction with the KEAP1 protein, thereby facilitating the nuclear translocation of Nrf2. Consequently, this process activates the Nrf2/ARE axis, contributing to the reduction of OS-induced NDDs (in vivo and clinical studies)	[109,110]
Curcumin (Polyphenol) 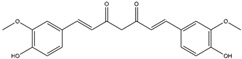	It is a bioactive compound naturally found in Indian spices and can be isolated from the roots of turmeric, a staple in Indian cuisine (*Curcuma longa* L.)	Curcumin can inhibit KEAP1, leading to the upregulation of the Nrf2/HO-1 axis, and concurrently activates the synthesis of the antioxidant peptide, glutathione. This mechanism has the potential to target α-synuclein and Aβ oligomers in PD and AD, respectively (in vitro study).	[112,113]
Zerumbone (Monocyclic Sesquiterpenoid) 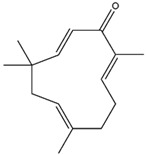	It is a bioactive compound found in perennial herbs and can be isolated from wild ginger (*Zingiber zerumbet* L.).	Zerumbone can activate the Nrf2/ARE axis, inducing antioxidant genes such as HO-1, GCLC, GCLM, and NQO1. This activation contributes to the alleviation of oxidative stress and neuroinflammation-induced NDDs (in vitro and in vivo studies)	[114,115]
Falcarinol (Polyacetylene) 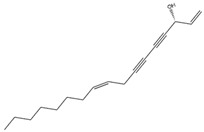	It is a bioactive compound found in a large number of plants of the Apiaceae family and can be isolated from carrots (*Daucus carota* L.).	Falcarinol can induce the Nrf2/ARE axis, leading to the activation of SOD and HO-1 enzymes. Consequently, this activation causes a reduction in cellular toxins such as LDH and MDA (in vivo study)	[116,117,118]
Nordihydroguaiaretic acid (Lignan) 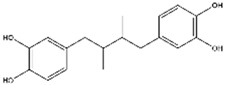	It is a bioactive compound and can be isolated from the evergreen desert shrub named creosote bush (*Larrea tridentata* (DC.) Coville).	Nordihydroguaiaretic acid can induce the Nrf2/HO-1 axis, which leads to reduced OS-induced NDDs (in vitro and in vivo studies)	[119,120]
Bixin (Apocarotenoid) 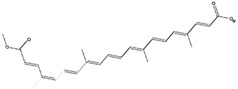	It is a natural pigment isolated from the seeds of achiote tree (*Bixa orellana* L.).	Bixin can induce the Nrf2/ARE axis, leading to the reduction of pro-inflammatory cytokines. This effect can contribute to the mitigation of neuroinflammation-induced NDDs (in vivo and clinical studies)	[121,122]
Withaferin A (Steroidal Lactone) Withanolides 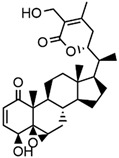	It is a bioactive compound with applications in herbal Indian medicine and can be isolated from the Indian winter cherry [*Withania somnifera* (L.) Dunal].	Withaferin A can induce the Nrf2/ARE axis through its interaction with the cysteine residue of KEAP1, thereby disrupting the KEAP1-Nrf2 complex. This mechanism has ameliorating effects on brain injury related to ALS (in silico, in vitro, and in vivo studies)	[123,124]
Epigallocatechin-3-gallate (EGCG) (Flavonoid) 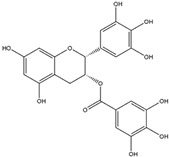	It is a potent antioxidant found abundantly in green tea and can be isolated from the fresh leaves of the tea plant [*Camellia sinensis* (L.) Kuntze].	EGCG can induce the Nrf2/ARE axis, leading to the upregulation of endogenous antioxidant genes such as *HO-1* and *GPx*. Additionally, it can bind to Aβ and α-synuclein, reversing the neurodegenerative process (in vivo and pre-clinical studies).	[125,126,127]
Rosmarinic acid (Polyphenol) 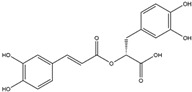	It is a bioactive compound found in the plant family of lamiaceae and can be isolated from rosemary (*Rosmarinus officinalis* L.).	Rosmarinic acid can induce the Nrf2/ARE axis, thereby eliciting a reduction in OS and subsequent cellular toxins. It can be a promising therapeutic agent in the context of NDDs (in vivo and clinical studies).	[128,129,130]
Myricetin (Flavonoid) 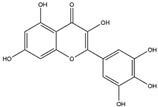	It is a bioactive compound found in the plants of the Polygonaceae family and can be isolated from birdweed (*Polygonum suffruticosum* Salzm. ex Ball).	Myricetin demonstrates the capability to induce the Nrf2/ARE axis, resulting in the upregulation of serum levels of antioxidants, including CAT, SOD, and HO-1. This can contribute to the detoxification of protein aggregates in the brain associated with NDDs (in vivo and preclinical studies).	[131,132,133]
Quercetin (Flavonoid) 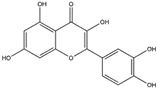	It is a bioactive compound found in a variety of plants. It can be isolated from sustainable onion skin/peel waste (*Allium cepa* L.).	Quercetin exhibits the ability to induce the Nrf2/ARE axis, resulting in the upregulation of antioxidant genes such as HO-1, NQO1, and SOD2, mitigating OS. This property positions quercetin as a potential alternative therapeutic agent against NDDs (in vivo and clinical studies).	[134,135,136]
Resveratrol (Polyphenol) 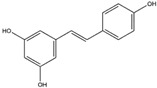	It is a polyphenolic compound found in nuts, berries, grapes, and red wine, and can be isolated from the skin of dark grapes (*Vitis vinifera L.*).	Resveratrol inhibits the KEAP1 protein, facilitating the translocation of Nrf2 to the nucleus, thereby activating the Nrf2/HO-1 axis. Furthermore, the downstream cascade of Nrf2 stimulates mitochondrial biogenesis and mitigates OS-induced NDDs (in vitro and in vivo studies).	[137,138,139]
Formononetin (Isoflavone) 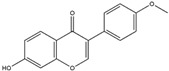	It is a naturally occurring compound found in legumes and can be isolated from red clover (*Trifolium pratense* L.).	The binding of formononetin to a specific domain of KEAP1 induces a conformational change, resulting in the liberation of Nrf2 from the Nrf2-KEAP1 complex. This, in turn, leads to the induction of the Nrf2/ARE axis, promoting redox homeostasis. Additionally, formononetin demonstrates the ability to remove Aβ plaques and inhibit MAO-B activity, contributing to the preservation of dopaminergic neurons and thereby preventing NDDs (in silico and in vitro studies).	[140,141,142]
Sinapic Acid (Hydroxycinnamic Acid) 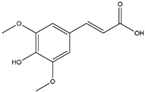	It is a phenolic acid with antioxidant properties, and is found in coffee, tea, and wine. It can be isolated from bokunja wine, which is made from Korean bramble (*Rubus coreanus* Miq.).	Sinapic acid induces the Nrf2/ARE axis, leading to the upregulation of antioxidant genes such as *GPx*, *CAT*, and *HO-1*. Simultaneously, it inhibits the AChE enzyme and blocks cholinergic receptors, thereby mitigating NDDs (in vitro and in vivo studies).	[143,144,145]
Chlorogenic Acid (Cinnamate Ester) 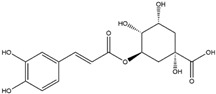	It is a polyphenolic compound found in a variety of green and roasted coffee cultivars. It can be isolated from *Coffea canephora* Pierre ex A. Froehner.	Chlorogenic acid induces the Nrf2/HO-1 axis, triggering downstream cascades that stimulate the NF-κB pathway. This cascade has the potential to reverse NDDs by modulating inflammatory processes (in vitro, in vivo, and clinical studies).	[146,147,148]
Ginkgo Extract (Flavonoids and Terpenoids, and Lignan) 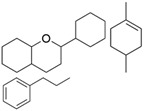	Derived from the leaves of the *Ginkgo biloba* L. tree.	Ginkgo extract inhibits KEAP1, activating the Nrf2/ARE axis and upregulating antioxidant enzymes such as SOD and CAT within the hippocampus. This crucial molecular mechanism has the potential to effectively reverse NDDs (in vivo study)	[149,150,151]
Indian Pennywort Extract (Saponin) 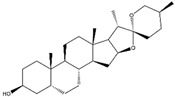	Extracted from Indian pennywort [*Bacopa monnieri* (L.) Wettst.].	Bacopa extract induces the Nrf2/ARE axis, activating antioxidant enzymes like HO-1 and GCLC. This molecular mechanism mitigates mitochondrial dysfunction and alleviates oxidative stress-induced NDDs.	[152,153,154]

AChE: acetylcholinesterase; MDA: malondialdehyde; NQO1: NAD(P)H quinone dehydrogenase 1; KEAP1: Kelch-like ECH-associated protein 1.

## Data Availability

All data supporting the findings of this study are available within the manuscript.
